# Dr. Boling on Quinine in Fevers and Inflammation

**Published:** 1844-10

**Authors:** 


*Dr. Boling on Quinine in Fevers and Inflammation.*—As to the best time for administering the quinine, I generally, in cases where there is anything like a distinct remission, prefer this period for its commencement, probably now, more from habit than anything else, where the remissions are short, or when the case is urgent, and there is reason to apprehend a fatal termination in the next exacerbation. Or where the disease is of so violent a character as to justify fears of the occurrence of any serious organic lesion, or a considerable aggravation of any that may already exist, it seems to me preferable to commence with it immediately, and this I generally do without regard to the stage of the paroxysm. As to the doses, they should be efficient; but very large ones are generally unnecessary, and they are only advisable where a severe exacerbation is expected, and the time allowed you to guard against it is short. To an adult, in a case in which its continued administration, for a length of time, is advisable, for the purpose of subduing local inflammation, by its sedative influence over the heart and arteries, about forty-eight grains in the twenty-four hours will generally be found sufficient; and it is better to give it in doses of about eight grains every four hours, than to administer it in smaller doses at shorter intervals. The length of time for which it should be continued varies according to the character of the case. In remittent fevers, in which any local affection that may be present,—e. g., the congestion of the brain in comatose remittent fever,—subsides entirely during remission, its continuance merely for a sufficient length of time to prevent an exacerbation is all that is necessary, the progress towards recovery, however slow, being almost always certain from this point; where, however, there is inflammation present, the remedy should be continued till it is entirely subdued; as frequently, when any spark of inflammatory action remains unextinguished, as soon as the influence of the quinine over the arterial system subsides, the inflammation is quickly rekindled, and its progress is generally very rapid.

The system once fully under the influence of the remedy, under any circumstances, it seems to me better *gradually* to withdraw it. Even in severe cases of congestive fever, where great prostration, restlessness, and anxiety exist,—where the pulse is very frequent, small, and weak, and the extremities bathed in a cold and clammy perspiration, and where each dose of the *so called* stimulant augments, in a high degree, these very symptoms which it is administered to combat,—I think that I have seen bad results follow its *sudden* withdrawal. In consultation, in such cases, I advise its continuance, the doses to be gradually diminished, but combined with full doses of some *genuine* stimulant.

From what I have said, it is apparent that I believe the beneficial influence of quinine, in such cases, to be excited by a property which it possesses, altogether distinct and different from its antiphlogistic or sedative action. I allude to its power of preventing the recurrence of the paroxysms of periodical diseases.

Its administration in congestive fever then, should be with the object solely of preventing an exacerbation, and thus allowing the efforts of nature to go on uninterrupted from the time of the decline of the previous paroxysm. With this object in view its administration can generally be conducted with safety, in the less severe cases, by giving no more than is necessary for this purpose, and then gradually withdrawing it; and in the more severe cases, where the prostration is extreme, by giving it in the same doses, with the same object only in view, in combination with some active stimulant to counteract its depressing influence. Thus, in the treatment of inflammatory affections, its sedative or contra-stimulant properties are aided by combining it with antimonials, and a general antiphlogistic course, while in cases of a congestive character its mysterious powers, as an agent against the periodical recurrence of morbid action, may be brought into play, and its depressing influence measurably prevented by combining it with stimulants.

It is where it has been given as a stimulant that I have seen its baneful influence manifested, in cases of great debility; and it is astonishing with what perseverance it is sometimes administered, while each dose is speedily followed by an increase in the exhausting perspiration, prostration, and weakness, and quickness of the pulse.

The *peculiar* applicability of the quinine to the treatment of the inflammatory affections of malarious districts, is owing to the combination, with its antiphlogistic properties, of a power as an agent in controlling the periodicity of morbid action. As an antiphlogistic remedy in elevated and healthy localities, it will probably never supercede the lancet, antimonials, &c., though it may, in many cases, be brought to their aid; but in malarious regions, ere long, it will generally be looked upon as the safest and most manageable contra-stimulant we possess, and at the same time one sufficiently powerful, while other agents of the same class will only be used to fulfil some casual indication, or as adjuvants to this, the principal remedy.—*Amer. Jour. Med. Sci.* for July, 1844.

